# Depolymerization and conversion of lignin to value-added bioproducts by microbial and enzymatic catalysis

**DOI:** 10.1186/s13068-021-01934-w

**Published:** 2021-04-03

**Authors:** Caihong Weng, Xiaowei Peng, Yejun Han

**Affiliations:** 1 National Key Laboratory of Biochemical Engineering, Institute of Process Engineering, Chinese Academy of Sciences, Beijing, 100190 China; 2 School of Chemical Engineering, University of Chinese Academy of Sciences, Beijing, 100049 China

**Keywords:** Lignin, Depolymerization, Enzymatic degradation, Lignin-derived aromatics, Metabolic pathways, Value-added bioproducts, Biosynthesis

## Abstract

Lignin, the most abundant renewable aromatic compound in nature, is an excellent feedstock for value-added bioproducts manufacturing; while the intrinsic heterogeneity and recalcitrance of which hindered the efficient lignin biorefinery and utilization. Compared with chemical processing, bioprocessing with microbial and enzymatic catalysis is a clean and efficient method for lignin depolymerization and conversion. Generally, lignin bioprocessing involves lignin decomposition to lignin-based aromatics via extracellular microbial enzymes and further converted to value-added bioproducts through microbial metabolism. In the review, the most recent advances in degradation and conversion of lignin to value-added bioproducts catalyzed by microbes and enzymes were summarized. The lignin-degrading microorganisms of white-rot fungi, brown-rot fungi, soft-rot fungi, and bacteria under aerobic and anaerobic conditions were comparatively analyzed. The catalytic metabolism of the microbial lignin-degrading enzymes of laccase, lignin peroxidase, manganese peroxidase, biphenyl bond cleavage enzyme, versatile peroxidase, and β-etherize was discussed. The microbial metabolic process of H-lignin, G-lignin, S-lignin based derivatives, protocatechuic acid, and catechol was reviewed. Lignin was depolymerized to lignin-derived aromatic compounds by the secreted enzymes of fungi and bacteria, and the aromatics were converted to value-added compounds through microbial catalysis and metabolic engineering. The review also proposes new insights for future work to overcome the recalcitrance of lignin and convert it to value-added bioproducts by microbial and enzymatic catalysis.

## Background

Converting the renewable biomass to chemicals and fuels is an attractive and green method for the sustainable environment development. Lignocellulose, the most abundant renewable resource in nature, is mainly composed of cellulose, hemicellulose, and lignin [1]. Cellulose and hemicellulose can be degraded into monosaccharides by enzymatic hydrolysis [2–5] and fermented to various bioproducts [6, 7], while most lignin cannot be utilized efficiently. Large amounts of lignin have been formed and estimated to be in the range 5–36 × 10^8^ tons annually [8]. Among them, the biomass refinery and pulp/paper industries contribute about 6.2 × 10^7^ and 5 × 10^7^ tons of lignin per year, respectively, including kraft lignin, lignosulfonate, and soda lignin [9]. In most cases, lignin is currently used for energy supply or discarded as waste. Lignin is a promising feedstock to produce biofuels and biochemicals owing to its high carbon-to-oxygen ratio and rich aromatic skeleton. To exploit lignin valorization, it is an urgent need to understand the degradation process and develop efficient metabolic pathway for conversion.

**Fig. 1:**
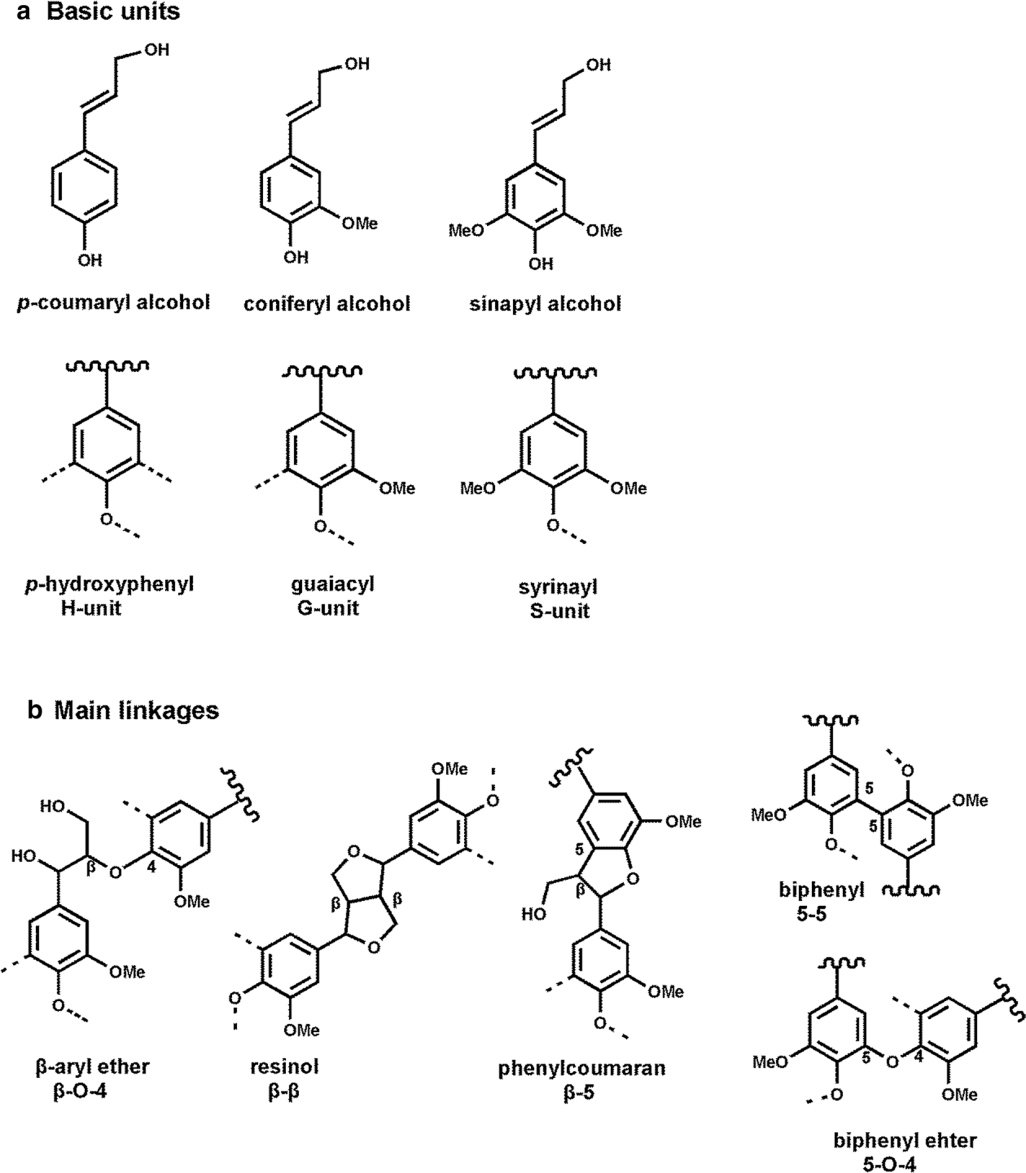
Major backbone units and representative linkages in lignin molecule. **a** The building blocks of lignin consist of three primary types of monolignols, namely *p*-coumaryl alcohol, coniferyl alcohol, and sinapyl alcohol. The alcohols form the corresponding phenylpropanoid units like p-hydroxyphenyl (H), guaiacyl (G), and syringyl (S) in lignin polymer, respectively. **b** Backbone units are conjugated via different chemical bonds (e.g., β-O-4, β-β, 5–5, and β-5) resulting in high resistance to lignin depolymerization

Lignin is an amorphous heteropolymer consisting of three phenylpropanoid units of guaiacyl alcohol, *p*-coumaryl alcohol, and syringyl alcohol, which are connected by the chemical bonds of aryl ether (β-O-4), phenylcoumaran (β-5), resinol (β-β), biphenyl ether (5-O-4), and dibenzodioxocin (5–5) [10] (Fig. 1). The complex structure and recalcitrance of lignin are the main challenges for its efficient depolymerization and utilization. Currently, thermochemical and biological approaches are the main methods for lignin depolymerization. Thermochemical processes including pyrolysis (thermolysis), gasification, hydrogenolysis, and chemical oxidation require stringent conditions, intensive energy input, and expensive facilities [11]. In contrast, the bioprocessing of lignin has the advantage of high specificity, low-energy input, and cost-effectiveness [12]. The bioprocessing treatment with microorganism normally include two steps: native lignin is firstly degraded to heterogeneous aromatics, which then enter the central carbon metabolism [13]. So far, a large number of microorganisms including fungi and bacteria have been found to be able to degrade and metabolize lignin.

Lignin is mainly depolymerized by extracellular oxidases secreted by microorganisms such as lignin peroxidase (LiP), manganese peroxidase (MnP), versatile peroxidase (VP), dye-decolorizing peroxidase (DyP), and laccase [14]. During the oxidation process, the unstable free radicals produced by the oxidase can attack lignin, and cleave the chemical bonds [15]. The resulted aromatic compounds were further metabolized by microbes via the enzymatic reactions [16] and β-ketoadipate pathway, and finally converted to valuable products. To now, lignin has been successfully manufactured into value-added products of polyhydroxyalkanoates (bioplastic) [17], lipids (often used as biofuel) [18], animal feed additive [19], pesticides [20], compost (generally as biofertilizers) [21], vanillin [22], and muconic acid [23].

In the present review, we focused on the bioprocessing of lignin and bioconverting it to value-added bioproducts. The most recent development on lignin depolymerization by microorganisms, the microbial secreted oxidases, and the decomposition mechanism were summarized. Additionally, the metabolism of lignin-derived aromatics in different microorganisms was illustrated and the production of value-added products through microbial metabolic engineering was proposed.

## Lignin depolymerization by microorganisms and enzymes

### Lignin depolymerization by fungi

The depolymerization of lignin is critical for lignin utilization, and diverse lignin-degrading enzymes and metabolic system of microorganism have been evolved for lignin degradation and conversion [24]. Fungi are the most effective lignin-degrading microorganisms, which can secrete a variety of lignin-degrading enzymes. According to the degradation mechanism of lignin, the lignin-degrading fungi mainly include three types: white-rot, brown-rot, and soft-rot fungi [25]. Among the three lignin-degrading fungi, only white-rot fungi can completely degrade lignin to CO_2_ and H_2_O [26], and the typical fungi for lignin degradation are shown in Table 1.

**Table 1:** Fungi degradation of lignin in various biomass sources

Microorganisms	Strains	Biomass materials	Lignin degradation	Ref
White-rot fungi	*Phanerochaete chrysosporium*	Wheat straw and cornstalk	30% and 34.3%	[31, 32] (Singh et al., Zhao et al.)
	*Pleurotus ostreatus*	Rice straw	41%	[154] (Taniguchi et al.)
	*Lentinula edode* LE16	Sugarcane bagasse	87.6%	[34] (Dong et al.)
	*Phlebia* sp. MG-60	Oak wood	40.7%	[35] (Kamei et al.)
	*Ceriporiopsis subvermispora*	Pinus taeda wood chips	22%	[36] (Guerra et al.)
	*Trametes versicolor*	Radiata pine wood chips	22%	[155](Shirkavand et al.)
	*Dichomytus squalens*	Wheat straw	34.1%	[44] (Knežević et al.)
Brown-rot fungi	*Gloeophyllum trabeum*	Wafers of spruce wood	16%	[42] (Yelle et al.)
	*Fomitopsis pinicola*	Wheat straw	32.4%	[44] (Knežević et al.)
	*Polyporus ostreiformis*	Rice straw	18.6%	[156] (Dey et al.)

*White-rot fungi* White-rot fungi are the main lignin degradation microorganism in nature, and its degradation ability is better than brown-rot and soft-rot fungi [25]. The lignin-degrading white-rot fungi include most strains of basidiomycetes and a few species of ascomycetes [27]. Among white-rot fungi, the species of *Ceriporiopsis subvermispora*, *Phellinus pini*, *Ganoderma australe*, and *Phlebia tremellosa* specifically degrade lignin and hemicellulose but not cellulose. However, other strains such as *Phanerochaete chrysosporium, Trametes versicolor*, *Heterobasidion annosum*, and *Irpex lacteusare* can simultaneously degrade cellulose, hemicellulose, and lignin [28, 29]. The main extracellular enzymes secreted by white-rot fungi for lignin-degrading were oxidases and peroxidases. The oxidative reactions catalyzed by oxidoreductase for lignin decomposition include the cleavage of carbon–carbon bonds and ether linkages, and the removal of side chain and aromatic rings [30].

*P. chrysosporium* is a model white rot fungus for lignin degradation, which has been applied for biological pretreatment of lignocellulosic biomass [31, 32]. The enzymes of MnP and LiP produced by *P. chrysosporium* degrade lignin in a non-specific oxidative way [33]. The fungi of *Lentinula edode* LE16 and *Pleurotus ostreatus* PO45 were also found to degrade lignin of sugarcane bagasse by producing polyphenol oxidase (PPO) and MnP [34]. Some white rot fungal species can produce laccase and peroxidases for lignin oxidation and decomposition. Lignin of oak wood was directly converted to ethanol by fermentation with *Phlebia* sp. MG-60, and MnP and laccase were identified in the culture [35]. *Subvermispora* was applied for *Pinus taeda* wood chips bioprocessing in solid-state fermentation, the lignin of which was mainly degraded by the β-O-aryl ether cleavage by the MnP and laccase [36]. The laccase (*Lcc1*) isolated from *Ganoderma tsugae* can promote lignin decomposition, mycelium growth, pigment formation, and stipe elongation [37]. The lignin degradation property of white-rot fungi makes it useful in the biopulping process of paper industry. In addition, white-rot fungi have been applied to other industrial fields: bioremediation of soil and water and biorefinery of biomass [38, 39].

*Brown-rot fungi and soft-rot fungi* Brown-rot fungi grow primarily on softwoods and represent 7% of wood-rotting basidiomycetes. The fungi can rapidly hydrolyze the component of cellulose and hemicellulose while just partially oxidize lignin. Brown-rot fungi were found to degrade lignin through hydroxyl radicals produced via Fenton oxidation chemistry [40]. The extracellular hydroquinones generated by brown-rot fungi can reduce Fe^3+^ of Fe–oxalate complex to Fe^2+^, which then reacts with hydrogen peroxide (H_2_O_2_) to generate hydroxyl radicals. The oxidized quinone can be converted to hydroquinone and achieve redox cycling [41].

*Gloeophyllum trabeum* can non-selectively break the intermonomer side-chain linkages of lignin, and its fermentation can cause 16% of lignin loss in spruce wood [42]. Yelle et al. [43] found that the content of the arylglycerol-β-aryl ether linkage of lignin decreased when aspen wood was treated with *Postia placenta*, which can produce an extracellular Fenton system and break lignin with hydroxyl radicals. It was identified that 32.4% of lignin was degraded by *Fomitopsis pinicola* after fermented for 2 weeks [44].

In addition to brown-rot fungi, soft-rot fungi can also degrade lignin by attacking the syringyl units [30]. Soft-rot fungi mainly include *Ascomycetes* and *Deuteromycetes* and preferentially degrade hardwood [45]. The soft-rot fungi *Aspergillus niger* and *Penicillium chrysogenum* were found to degrade pine and sycamore wood [46], and some soft-rot fungi can degrade vanillic acid and phenols rapidly [47]. While little is known about the enzymes of soft-rot fungi involved in degrading lignin, it was suggested that soft-rot fungi might modify rather than mineralize lignin.

### Lignin depolymerization by bacteria

Apart from fungi, bacteria with lignin degradation ability have been identified from different habitats such as soil, rotten wood, wastewater treatment plant, and animal gut [48]. Although the lignin degradation performance of bacteria is inferior to fungi, bacteria have stronger environmental adaptability. Recent studies reported that *Actinobacteria*, *Proteobacteria*, and *Firmicutes* are major lignin-degrading bacteria [49]. Bacteria grow on lignin secrete oxidative enzymes to break lignin with the presence of oxygen. Moreover, lignin can be degraded by extreme anaerobic conditions, and the bacteria with capability of decomposing lignin are listed in Table2.

**Table 2:** Bacteria degradation of lignin in various biomass sources

Microorganisms	Strains	Biomass materials	Lignin degradation	Ref
Aerobic bacteria	*Streptomyces viridosporus* T7A	Softwood spruce, hardwood maple and grass	30.9%, 32%, and 44.2%	[52] (Antai*,* Crawford)
	*Rhodoccocus Jostii* RHA1	Soluble and lignin-rich stream	18.9%	[55] (Salvachúa et al.)
	*R. pyridinivorans* CCZU-B16	Alkali lignin	30.2%	[128] (Chong et al.)
	*Pseudomonas putida* KT2440	Alkaline pretreated liquor	~ 30%	[58] (Salvachúa et al.)
	*P. putida* NX-1	Kraft lignin	28.5%	[59] (Xu et al.)
	*Comamonas* sp. B-9	Kraft lignin	45%	[61] (Chai et al.)
	*Bacillus ligniniphilus* L1	Alkaline lignin	38.9%	[62] (Zhu et al.)
	*B. amyloliquefaciens* SL-7	Tobacco straw lignin	28.55%	[63] (Mei et al.)
Facultative anaerobe bacteria	*Enterobacter lignolyticus* SCF1	Alkali lignin	56%	[68] (DeAngelis et al.)
	*Acetoanaerobium* sp	Kraft lignin	24.9%	[71] (Duan et al.)
Extremophile bacteria	*Caldicellulosiruptor kronotskyensis*	Natural rice straw	52.5%	[74] (Peng et al.)
	*Arthrobacter* sp. C2	Sodium lignin sulfonate	40.1%	[75] (Jiang et al.)

*Aerobic bacteria* Bacterial lignin depolymerization primarily occurs under aerobic conditions [50]. *Streptomyces* and *Rhodoccocus* of *Actinobacteria* are typical bacteria for lignin degradation. *Streptomyces viridosporus* T7A decompose lignin by extracellular enzymes secreted by filamentous form [13]. *S. viridosporus* T7A can degrade lignin of native wheat straw, and the guaiacyl units reduced [51]. Approximately 30 ~ 45% of the lignin from softwood, hardwood, and grass was removed after fermentation with *S. viridosporus* T7A and *S. setonii* 75Vi2 for 12 weeks [52]. *Rhodoccocus* is considered as a robust microorganism for lignin breakdown, as it has excellent tolerance and hydrolytic activity for toxic metabolites. The polychlorinated biphenyl-degrading soil bacterium *R. jostii* RHA1 can convert kraft lignin and wheat straw to aromatic dicarboxylic acids and vanillin, and around 19% of lignin can be utilized [53–55]. The dyp-type peroxidase DypB from *R. jostii* RHA1 was identified to break β-aryl ether linkage in lignin model compound [56]. *R. erythropolis* isolated from wood and soil also showed high degrading activity on nitrated-lignin of wheat straw [57]. The *Proteobacteria* containing *Pseudomonas, Pandoraea,* and *Comamonas* genus, etc. were applied for lignin depolymerization. It was found that ~ 30% lignin of alkaline pretreated liquor (APL) were depolymerize and catabolize by *P. putida* KT2440 and *P. putida* mt-2 [58]. *P. putida* NX-1 could utilize kraft lignin as the sole carbon source for cell growth and secrete extracellular ligninolytic enzymes [59]. *P. putida* is an excellent chassis bacterium for converting lignin-derived aromatics to bio-based products through metabolic engineering. *Pandoraea* sp. B-6 was also found to efficiently degrade kraft lignin and produce low-molecular-weight aromatic and acid-type compounds [60]. The decolorization and depolymerization of kraft lignin by *Comamonas* sp. B-9 were 54% and 45%, respectively, after 7 days of treatment [61]. *Bacillus* genus of *Firmicutes* with lignin-degrading ability was identified through high-throughput sequencing. Fifteen kinds of phenol ring aromatic compounds were generated from alkaline lignin processed with *Bacillus ligniniphilus* L1 [62]. Three kinds of lignin degradation pathways of gentisate, benzoic acid, and β-ketoadipate were identified in *Bacillus* through genomic and proteomic analysis. *Bacillus amyloliquefaciens* SL-7 can grow on tobacco straw lignin and secreted ligninolytic enzymes [63].

*Anaerobic bacteria* In addition to aerobic bacteria, anaerobic bacteria have been identified to convert lignin and its derived aromatics to methane and carbon dioxide. The degradation performance of modified lignin under anaerobic conditions is better than that of natural lignin, which has a high degree of methoxylation [64]. The methoxy group is the main attacking point for many bacteria during anaerobic degradation of methoxylated aromatics. The anaerobic process of lignin degradation mainly includes demethoxylation, aromatic ring cleavage, and methanogenesis [65]. Lignin-derived aromatics was converted to methane by anaerobically digestion by methanogenic microbial communities [66]. Many bacteria have been identified to be capable of degrading lignin in anaerobic environments. A facultative anaerobe *Enterobacter lignolyticus* SCF1 was isolated from tropical forest soil with alkali-treated lignin as the sole carbon source [67]. The genes of catalase/peroxidase and glutathione S-transferases for lignin degradation via 4-hydroxyphenylacetate pathway were up-regulated by transcriptomic and proteomic analyses [68]. *Tolumonas lignolytica* BRL6-1 and *Klebsiella* sp. strain BRL6-2 were isolated and characterized as anaerobic lignin-degrading bacteria, and several putative enzymes for lignin degrading were identified [69, 70]. *Acetoanaerobium* sp. WJDL-Y2 was identified from the sludge of a pulp and paper mill, which can oxidize kraft lignin to low-molecular-weight aromatic and acid compounds such as syringic acid, ferulic acid, and hexanoic acid [71].

*Extremophile bacteria* With the special enzymes and metabolic pathways, the extremophile bacteria were more competitive for lignin degradation and utilization. The thermophilic bacteria showed promising potential for the degradation and transformation of lignin. The Dyp-type peroxidase from *Thermobifida fusca* can degrade kraft lignin and oxidize a β-aryl ether lignin model compound [72]. When the hardwood of *Populus trichocarpa* was processed with the anaerobic thermophile *Clostridium thermocellum,* the β-O-4 linkage content was reduced and syringyl/guaiacyl (S/G) ratio in lignin was increased [73]. The extremely thermophilic bacterium *Caldicellulosiruptor kronotskyensis* can degrade natural rice straw without pretreatment, and produce solubilized carbohydrates, organic acids, and lignin-derived aromatics [74]. Besides, lignin degradation was also detected by a psychrotrophic bacteria *Arthrobacter* sp. C2 at low temperature, and the intermediates of acids, phenols, aldehydes, and alcohols were identified after the treatment [75].

## Lignin depolymerization by enzymes

The microbial degradation of lignin was conducted by a serious of oxidative enzymes. An increasing number of lignin-degrading enzymes have been discovered and applied in the processes of lignin depolymerization and mineralization from fungi and bacteria As the major enzymes for lignin degradation, phenol oxidase (laccase) and heme-containing peroxidases (lignin peroxidase, manganese peroxidase, and versatile peroxidase) have attracted considerable attention [76]. The in vitro enzymatic synthesis has been applied for lignin conversion, which avoids the cell culture, obstacle of substrate transport, and NAD(P)H and ATP imbalance [77]. The properties of typical ligninolytic enzymes are listed in Table 3, and the reactions and catalytic mechanisms were discussed.

**Table 3:** Characteristics and reaction of major ligninolytic enzymes [157, 158]

Enzyme	Source	Substrate	General reaction
Laccase EC 1.10.3.2	Widely distributed in fungi and bacteria (e.g., *Ascomycetes, Basidiomycetes* and *Streptomyces*)	Phenolic compounds, aromatic amines and dye molecules	4 benzenediol + O_2_ ⇌ 4 benzosemiquinone + 2H_2_O
Lignin peroxidase EC 1.11.1.14	White rot fungal genera (e.g., *Bjerkandera*, *Phanerochaete*, *Phlebia* and *Trametes*)	Phenols, aromatic amines, aromatic ethers and polycyclic aromatics	1,2-bis(3,4-dimethoxyphenyl)propane-1,3-diol + H_2_O_2_ ⇌ 3,4-dimethoxybenzaldehyde + 1-(3,4-dimethoxyphenyl)ethane-1,2-diol + H_2_O
Manganese peroxidase EC 1.11.1.13	Wood and litter-decomposing white rot fungi (e.g., *Dichomitus squalens*, *Agaricus bisporus* and *Agrocybe praecox*)	Phenolic compounds	2Mn(II) + 2H^+^ + H_2_O_2_ ⇌ 2Mn(III) + 2H_2_O
Versatile peroxidase EC 1.11.1.16	White rot species (e.g., *Pleurotus ostreatus*, *Bjerkandera adusta*)	High-redox-potential aromatic compounds and recalcitrant dyes	(1) Reactive Black 5 + H_2_O_2_ ⇌ oxidized Reactive Black 5 + 2 H_2_O (2) Donor + H_2_O_2_ = oxidized donor + 2 H_2_O
Dye-decolorizing peroxidase EC 1.11.1.19	Fungi and bacteria (e.g., *Ascomycetes*, *Basidiomycetes* and *Bacillus*)	Dye compounds, carotenoids and phenolics	Reactive Blue 5 + 2 H_2_O_2_ ⇌ phthalate + 2,2′-disulfonyl azobenzene + 3-[(4-amino-6-chloro-1,3,5-triazin-2-yl)amino] benzenesulfonate + 2 H_2_O

### Laccase

**Fig. 2:**
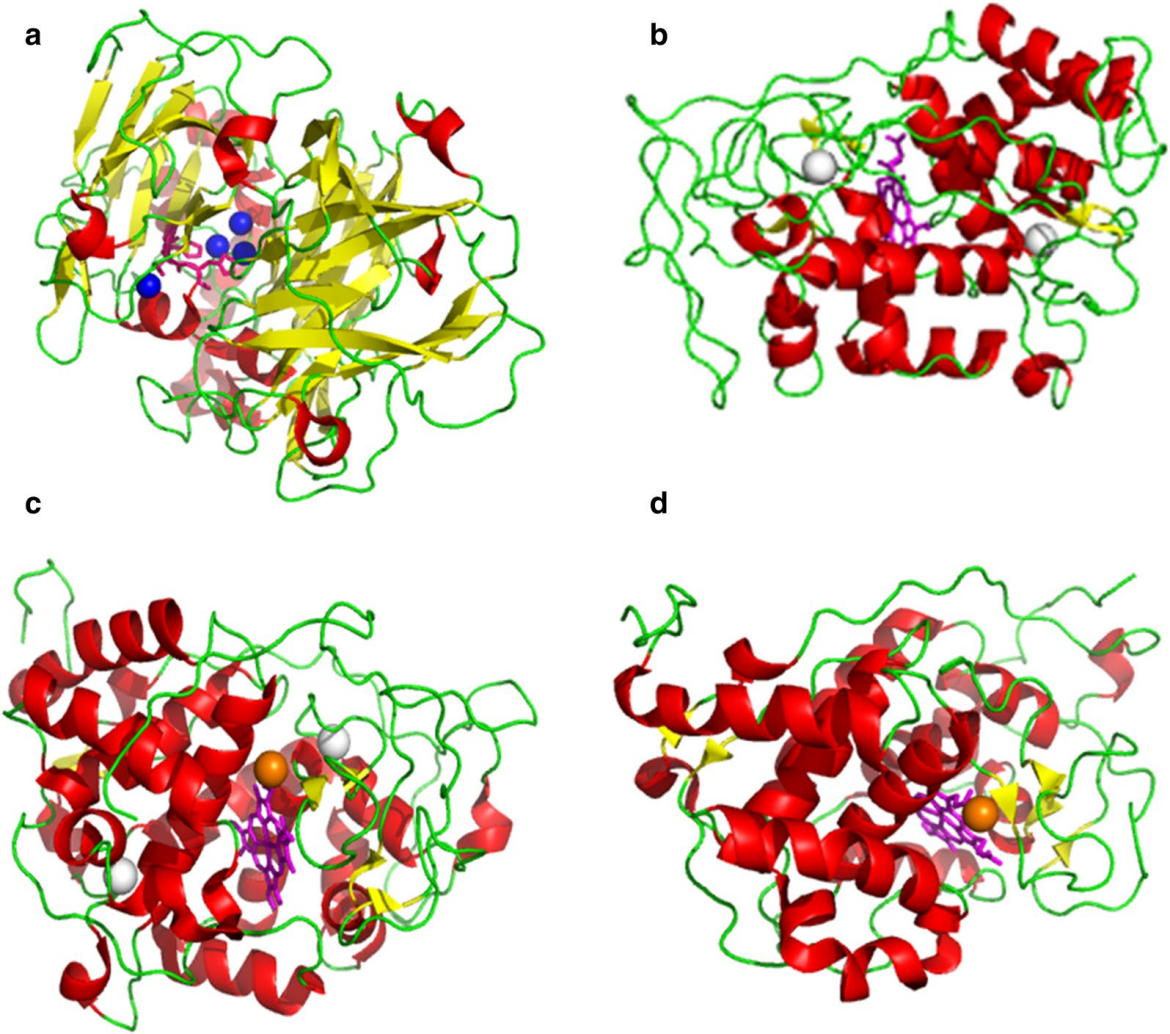
Structures of ligninolytic enzymes. The red, yellow, and green colored regions represent α-helix, β-sheet, and random coil, respectively. **a** Laccase (PDB ID: 1GYC) from *Trametes versicolor* [78] has a well-conserved active site with four copper, and T1 copper is connected to the trinuclear cluster by a His-Cys-His tripeptide. **b** Lip (PDB ID: 1LGA) of *Phanerochaete chrysosporium* [85] contains two calcium ions, four disulfide bonds, and a heme-containing one iron atom as its active site. **c** MnP (PDB ID: 1YYD) from *P. chrysosporium* [89] shows the active sites of Glu35, Glu39, and Asp179 residues as well as the Mn^2+^ ion. **d** VP (PDB ID: 2BOQ) of *Pleurotus eryngii* [94] exhibits an Mn^2+^-binding site and an external Trp residue. The electron transfer pathway towards heme is obtained directly from Mn^2+^ or relatively long range from Trp

**Fig. 3:**
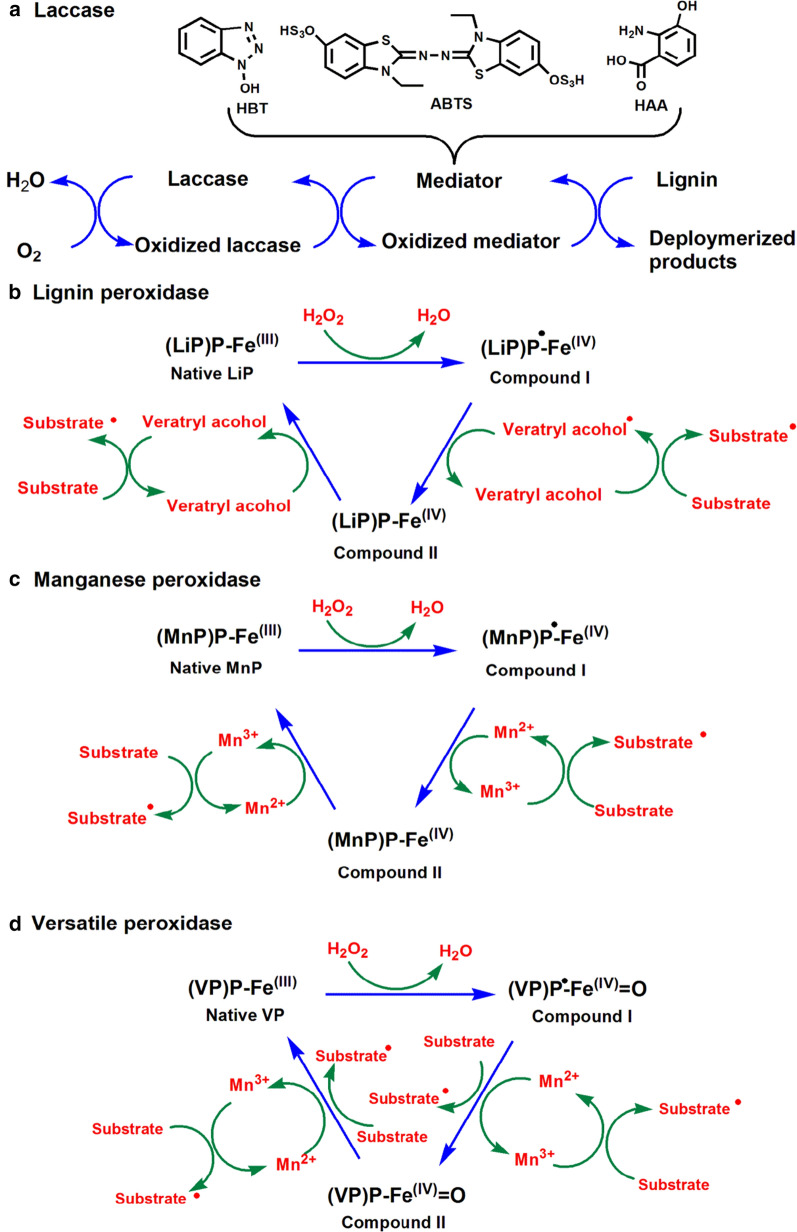
Catalytic mechanism of ligninolytic enzymes mediated lignin degradation. **a** Laccases not only directly oxidize phenolic compounds, but also degrade non-phenolic substrates of lignin in the presence of chemical mediators [79]. Molecular oxygen is reduced into water. **b** LiP indirectly degrades lignin via oxidizing veratryl alcohol to the corresponding diffusible cation radical as a direct oxidant on lignin. Two electrons of the native ferric enzyme are oxidized by H_2_O_2_ to form compound one, which receives one electron to form compound two. Finally, compound two is returned to the resting native ferric state by gaining one more electron from the reducing substrate [86, 87]. **c** MnP oxidizes the one-electron donor Mn^2+^ to Mn^3+^, which in turn oxidizes a large number of phenolic substrates. The native ferric enzyme initially reacts with H_2_O_2_ to form compound one, and an Mn^2+^ ion donates one electron to the porphyrin intermediate to form compound two. The native enzyme is similarly produced from compound two by obtaining one electron from Mn^2+^ [90, 91]. **d** The basic catalytic cycle of VP is similar to the MnP and LiP with the two intermediary compounds one and two

Laccase is a multi-copper oxidase present in fungi, plants, and bacteria, and the fungal laccase usually has higher reduction potential than that of plants and bacteria. The structure of laccase from *Trametes versicolor* has been characterized (Fig. 2a) [78]. It contains approximately 500 amino acid residues and three copper sites: type 1 (one Cu atom), type 2 (one Cu atom), and type 3 (two Cu atoms) per molecule of laccase. In the reaction catalyzed by type1 laccase, four electrons are transferred to the tri-nuclear center via a His–Cys–His tripeptide pathway. Both phenolic and non-phenolic compounds can be degraded by laccase with oxygen as a final electron acceptor (Fig. 3a) [79]. The oxidation of phenolic substrates by laccase forms phenoxyl free radical as an unstable intermediate, which then promotes Cα oxidation, alkyl-aryl cleavage, and Cα-Cβ cleavage [80]. Laccase needs to cooperate with the mediators like 1-hydroxybenzotriazole (HBT), 3-hydroxyanthranilic acid (HAA), and 2, 2′-azinobis-(3-ethylbenzothiazoline-6-sulfonate) (ABTS) in degrading non-phenolic substrates. The oxidized non-phenolic compounds coupled with mediators can promote the aromatic ring cleavage, Cα-Cβ cleavage, Cα oxidation, and β ether cleavage [81]. It is generally agreed that mediators enhance the oxidation capabilities of laccase and help overcome the steric hindrance existing between laccase and substrate. With the help of these mediators, laccase also can be applied in delignification process. The laccase of *Trametes villosa* could remove about 48% and 32% of lignin, respectively, from *Eucalyptus globulus* and *Pennisetum purpureum* feedstocks with 2.5% HBT as a mediator [82]. Different methods have been carried out to improve laccase yield of microbes including novel fermentation methods, genetic modifications, and addition of cofactors or inducer. Previous studies also found that the microbes can still degrade lignin in the absence of laccase, which suggested that laccase is the essential enzyme for lignin depolymerization [83, 84].

### Lignin peroxidase (LiP)

LiP is a glycoprotein with molecular mass of 38–43 kilodalton (kDa) and isoelectric point (pI) of 3.3–4.7 [76]. The crystal structure of LiP in *P. chrysosporium* was mainly constituted of α-helices (Fig. 2b) [85], and there are two calcium ions and four disulfide bonds to stabilize the three-dimensional structure. The active site of LiP is composed of a heme-containing iron atom. The trp171 residue conserved in LiP sequences is essential for the catalytic activity of LiP. LiP oxidizes both non-phenolic and phenolic compounds with H_2_O_2_ and veratryl alcohol (VA) as electron donor and cofactor (Fig. 3b). Generally, the enzymatic reaction of LiP-mediated lignin degradation consists of one oxidation and two reduction steps. The oxoferryl iron porphyrin radical cation [Fe(IV)=O^+^] is formed by the oxidation of ferric [Fe(III)] LiP along with the reduction of H_2_O_2_ to water. Then, [Fe(IV)=O^+^] was converted to two [Fe(IV)=O] through two consecutive one-electron reduction steps and complete catalytic cycle [86, 87]. LiP can degrade a variety of phenolic and nonphenolic compounds, and hence, it is a candidate for lignin depolymerization. Compared to other peroxidases, LiP is the major enzyme responsible for lignin degradation due to its high reduction potential. It was found that LiP produced in liquid-state fermentation of *Aspegillus oryzae* CGMCC 5992 showed high activity on lignin of corn stover pretreated with H_2_O_2_ [88], and the addition of mineral nutrients and gene modification were conducted to enhance LiP yield.

### Manganese peroxidase (MnP)

MnP is a glycosylated heme-protein with a molecular weight of 45 ~ 60 kDa [76], and it is the main ligninolytic peroxidase of basidiomycetes. The crystal structure of MnP from *P. chrysosporium* was published and presents similarities to Lip (Fig. 2c) [89], and it includes an Mn^2+^ ion, one heme propionate, and the side chains of Glu35, Glu39, and Asp179. The lignin degradation catalyzed by MnP includes both oxidation and reduction steps (Fig. 3c). MnP initiates the catalytic cycle by binding H_2_O_2_ to the native ferric enzyme. Afterward, MnP oxidizes Mn^2+^ to Mn^3+^ in the presence of chelators, and the generated Mn^3+^ then convert lignin phenolic compounds to phenoxy-radicals. The organic acid chelators like oxalate and malonate can stabilize Mn^3+^ and stimulate the enzyme activity [90, 91]. Similar to LiP, MnP plays an important role in the initial depolymerization of lignin. Moreover, it was found that adding MnP to the culture medium can accelerate lignin depolymerization. It was found that MnP can promote lignin degradation and methane yield, and 68.4% of lignin from municipal solid waste was removed by MnP [92].

### Versatile peroxidase (VP)

VP is a unique lignin-degrading enzyme and is found in white-rot fungal genera *Pleurotus* and *Bjerkandera* [93]. The crystal structure of VP from *Pleurotus eryngii* is similar to LiP and MnP of *P. chrysosporium* (Fig. 2d) [94]. An Mn^2+^-binding site was found in the protein structure allowing a direct transfer of electrons to the heme. And a tryptophan residue revealed the possibility of long-range electron transfer to oxidate aromatic compounds at the protein surface. VP has a broad substrate preference as containing a heme access channel, a catalytic tryptophan, and an Mn oxidation site. VP is termed as hybrid peroxidase, which exhibits similar catalytic mechanisms with both LiP and MnP (Fig. 3d). However, VP can degrade directly high reduction potential substrates without the presence of VA and oxidizing Mn^2+^ independently, which is different from MnP and LiP [95]. VP has obtained research interests in biotechnological applications and genetic manipulations due to its special bifunctionality. The VP of *Physisporinus vitreus* was used to reduce the saccharification recalcitrance and improve the enzymatic hydrolysis of corn stover [96]. The VP of *Bjerkandera adusta* was cloned and over-expressed in *Escherichia coli* for the large-scale production [97].

### β-Etherase

Besides peroxidase, β-etherases have been discovered to degrade lignin fragments in vivo, which could break β-aryl ether and biphenyl linkages within lignin molecules. The β-etherases involved in β-O-4 ether and biphenyl catabolic pathways provide a reliable method to depolymerize and convert lignin.

**Fig. 4:**
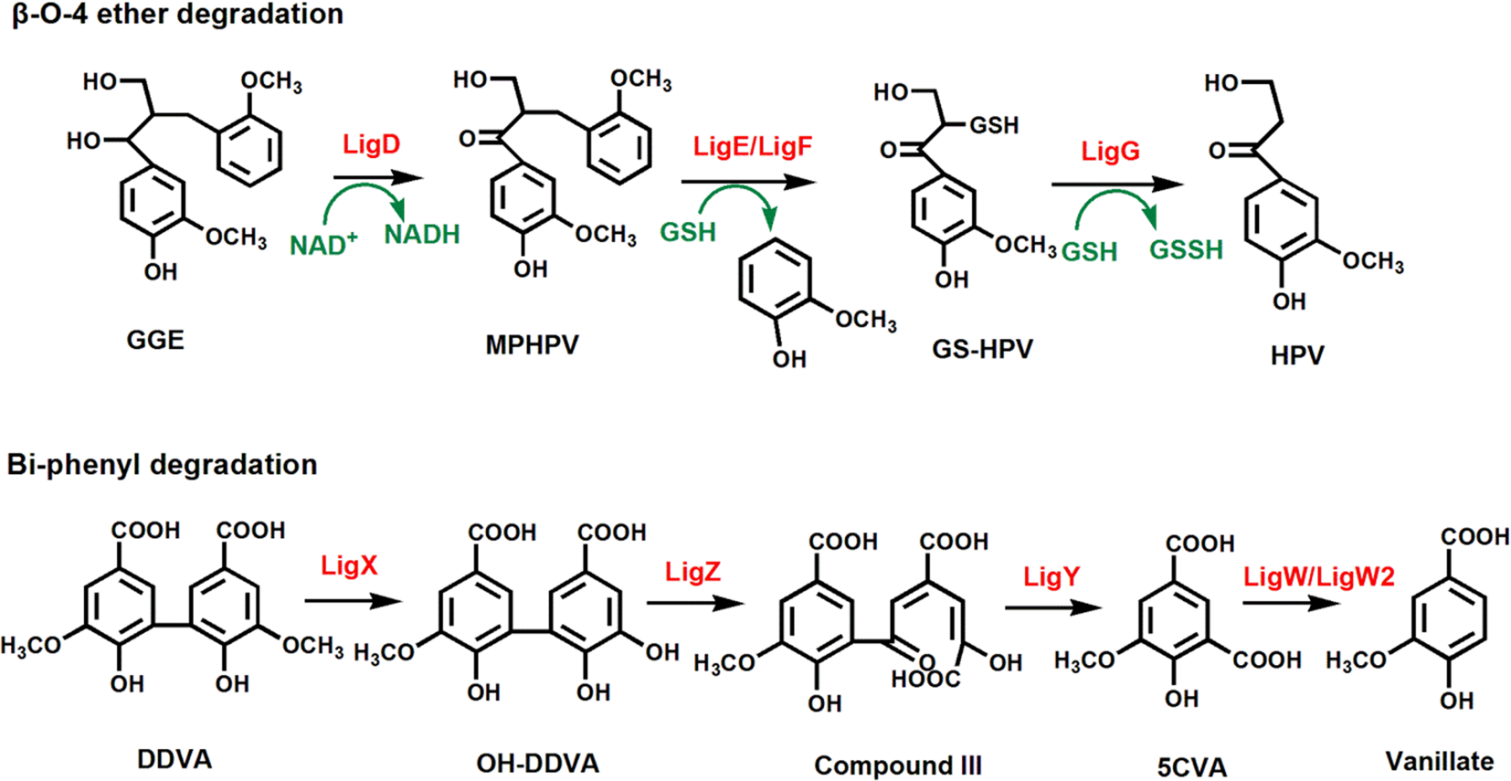
Mechanisms of β-O-4 ether and biphenyl linkage degradation. The enzymes involved in the breakdown of β-O-4 aryl ether and biphenyl bonds in bacteria are identified and characterized. In β-O-4 ether degradation, guaiacylglycerol-β-guaiacylethe (GGE) is first degraded to α-(2-methoxyphenoxy)-β-hydroxypropiovanillone (MPHPV), which is then converted to α-glutathionyl-HPV (GS-HPV) and β-hydroxyproppiovanillone (HPV) by LigD (Cα-dehydrogenase), LigE/LigF (β-etherase), and LigG (glutathione lyase) [102]. In biphenyl degradation, 5, 5′-dehydrodivanillate (DDVA) is initially *O*-demethylated to form 2,2′-3-trihydroxy-3′-methoxy-5,5′-dicarboxybiphenyl (OH-DDVA) by LigX (non-heme iron-dependent demethylase enzyme). The generated substrate is oxidated and cleaved by LigZ (extradiol dioxygenase) and LigY (C–C hydrolase) to produce 5-carboxyvanillic acid (5CVA). Eventually, 5CVA is transformed into the central intermediate vanillic acid by LigW and LigW2 (two decarboxylase enzymes) [106, 108]

The β-O-4 ether bond is the most prevalent linkage and accounts for more than 50% of all ether linkages in lignin [98], so its breakdown is critical for lignin depolymerization. Recently, the enzymatic cleavage of β-O-4 ether bond (Fig. 4) was studied in bacteria of *Sphingobium* sp. SYK-6, *Novosphingobium* sp. PP1Y, and *Dichomitus squalens* [99–101]. The degradation of β-O-4 ether bond starts with Cα-dehydrogenase LigD that oxidizes the hydroxyl group at Cα position with the consumption of NAD^+^. Subsequently, β-etherase LigE or LigF cleaves the intermediate to α-glutathionyl-β-hydroxypropiovanillone (GS-HPV) with glutathione at its Cβ position. While the glutathione is oxidized to glutathione (GSSG) by glutathione lyase LigG and releases the final product of β-hydroxyproppiovanillone (HPV) [102]. The enzymes of LigD, E, F, and G were crucial for lignin degradation, and the complete set of genes LigD, LigF, and LigG were expressed heterologously in *Arabidopsis thaliana* to cleave β-O-4 aryl ether bond, which enhanced lignin digestibility [103].

### Biphenyl bond cleavage enzyme

Biphenyl linkage is another major bond and makes up approximately 10% in softwood lignin [104]. Biphenyl linkages are also exist polychlorinated biphenyls (PCB), which are important environmental pollutants and carcinogens [105], and the degradation process of PCB has been extensively studied (Fig. 4). In the catalytic process of 5, 5′-dehydrodivanillate (DDVA) by non-heme iron-dependent demethylase enzyme LigX, one methoxy group is initially removed to form a hydroxyl group [106]. The product of LigX is the substrate for oxidative meta-cleavage via the extradiol dioxygenase LigZ [107]. Then, the C–C hydrolase LigY convert the ring fission product to 4-carboxy-2-hydroxypentadienoic acid and 5-carboxyvanillic acid (5CVA). Finally, the decarboxylases LigW and LigW2 transform 5CVA to the metabolic central intermediate vanillic acid or vanillate for the synthesis of bioproducts [108]. The cleavage of bi-phenyl linkage has been proved that it can promote lignin degradation.

## Biodegradation of lignin-derived aromatic compounds

**Fig. 5:**
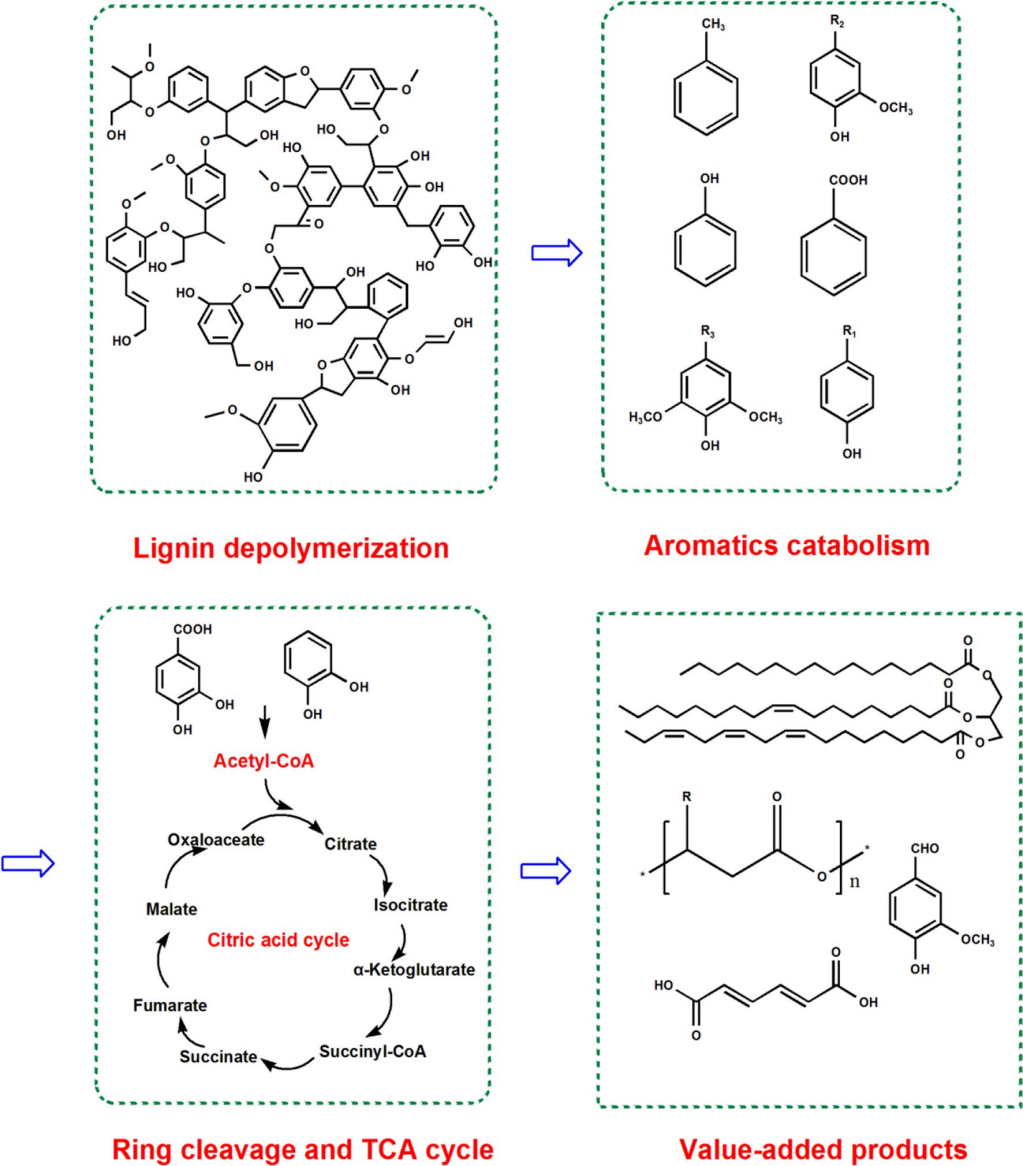
General route of lignin valorization via biochemical system. Lignin is first depolymerized via microorganisms and funneling various lignin-derived aromatics into protocatechuate and catechol. The central intermediates can be eventually converted to value-added compounds via aromatic ring cleavage and TCA cycle

Lignin depolymerization by microorganisms yields a heterogeneous mixture of low-molecular-weight aromatic compounds, which have certain toxicity and inhibit the growth of microorganisms. In nature, several bacteria have been reported to use the lignin-derived aromatics as carbon and energy sources for cell growth and value-added products accumulation [109]. Biological lignin degradation usually include three stages: lignin depolymerization, aromatics catabolism, and ring cleavage, the carbon of aromatic compounds ultimately integrates into TCA cycle (Fig. 5). The degradation of lignin produces three categories of monolignols (G-lignin, H-lignin, and S-lignin), which can be assimilated by various bacteria through different metabolic pathways.

### Degradation of H-lignin-based derivatives

**Fig. 6:**
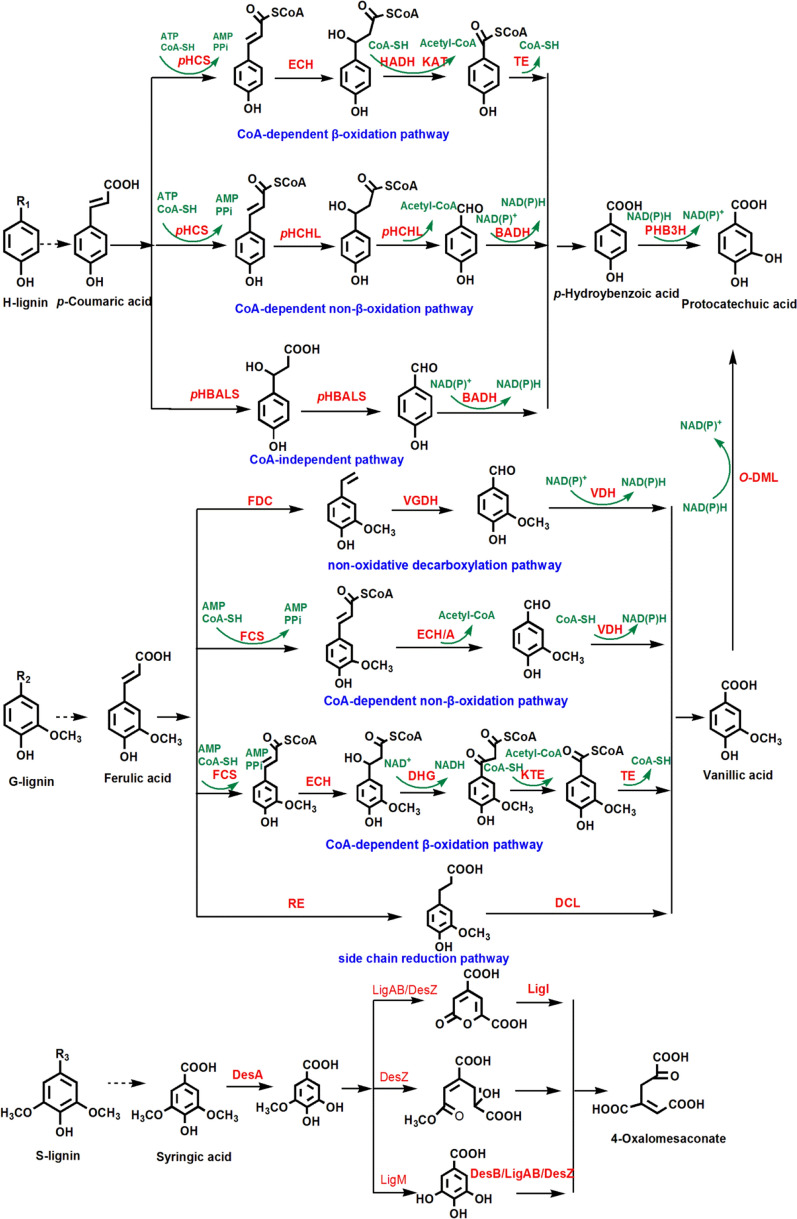
Degradation pathways for lignin-based aromatic compounds. H-lignin (*p*-coumaric acid) can be degraded to protocatechuic acid through three different pathways: CoA-independent pathway, CoA-dependent non-β-oxidation pathway, and CoA dependent β-oxidation pathway [111]. The degradation pathway of G-lignin (ferulic acid) can be divided into non-oxidative decarboxylation pathway, CoA-dependent non-β-oxidation pathway, CoA-dependent β-oxidation pathway, and side chain reduction pathway [118]. These four pathways are all transformed into vanillic acid involved with different intermediates and enzymes. S-lignin (syringic acid) is assimilated into 4-oxalomesaconate derived from the protocatechuic acid 4, 5-cleavage pathway via a series of enzyme reactions [120]

The H-lignin-based derivatives (*p*-coumaric acid) possess simple structure and low lignin content, which accounts for 0.3%, 1.7%, 2.8%, and 2.8% of the lignin in poplar (hardwood), pine (softwood), corn (monocotyledon), and *Arabidopsis* (dicotyledon), respectively [110]. Generally, the degradation pathways of *p*-coumaric acid in bacteria can be categorized as CoA-dependent β-oxidation pathway, CoA-dependent non-β-oxidation pathway, and CoA-independent pathway [111]. The generated intermediate of 4-hydroxybenzoate was hydroxylated to protocatechuic acid by 4-hydroxybenzoic acid-3-hydroxylase (Fig. 6). Among the three pathways, the CoA-dependent non-β-oxidation pathway of *p*-coumaric acid mainly occurs in *Rhodococcus* sp., *Sphingomonas* sp., and *Sphingobium* sp. [112–114]. *Burkholderia glumae* BGR1 was identified to catabolize *p*-coumaric acid via CoA-dependent non-β-oxidation pathway, the *p*-coumaric acid was converted to *p*-hydroxybenzaldehyde by *p*-hydroxycinnamoyl CoA synthetase (pHCS) and *p*-hydroxycinnamoyl CoA hydratase/lyase (pHCHL), and then oxidized to *p*-hydroxybenzoic acid by benzaldehyde dehydrogenase (BADH). The generated *p*-hydroxybenzoic acid undergoes a hydroxylation reaction to form protocatechuic acid [115]. Besides the protocatechuate pathway, new gentisate pathway for 4-hydroxybenzoate metabolism has been found in *Haloarcula* sp. strain D1, *Bacillus ligniniphilus* L1, and *Candida parapsilosis* [62, 116, 117]*.*

### Degradation of G-lignin-based derivatives

The G-lignin-based derivatives (ferulic acid) have a methoxy group on the aromatic ring, which constitutes 37.8%, 98.3%, 38.3%, and 77.1% of the lignin in poplar, pine, corn, and *Arabidopsis* respectively [110]. Ferulic acid can be converted to the intermediate vanillic acid rather than 4-hydroxybenzoate, through four different metabolic pathways of non-oxidative decarboxylation pathway, CoA-dependent β-oxidation pathway, CoA-dependent non-β-oxidation pathway, and side chain reduction pathway [118]. Under the catalysis of vanillate demethylase, vanillic acid is demethylated and transformed to protocatechuic acid (Fig. 6). *Pseudomonas fluorescens* BF13 and *Pseudomonas putida* KT2440 can degrade ferulic acid via CoA-dependent non-β-oxidation pathway. Ferulic acid is catalyzed to feruloyl-CoA by feruloyl-CoA synthetase (*fcs*), and then converted to vanillin and acetyl-CoA by enoyl-CoA hydratase/aldolase (*ech*). Finally, the vanillin dehydrogenase (*vdh*) oxidizes vanillin to vanillic acid, which is further decomposed to protocatechuic acid by vanillate-O-demethylase (*vanAB*) [119]. The understanding of metabolic pathways in related strains will help to increase the yield of products from ferulic acid through metabolic engineering.

### Degradation of S-lignin-based derivatives

The S-lignin-based derivatives (syringic acid) contain two methoxy groups in its aromatic ring, which makes them more difficult to be degraded than G- and H-lignins. The S-lignin occupies 61.9%, 0, 58.9%, and 20.1% of the lignin in poplar, pine, corn, and *Arabidopsis*, respectively [110]. Compared with ferulic acid and *p*-coumaric acid, only a few microbes such as *Sphingomonas* sp. SYK-6 strain can metabolize syringic acid [120]. The demethylation of syringic acid is catalyzed to 3-*O*-methylgallate (3MGA) by tetrahydrofolate-dependent *O*-demethylase (DesA). The produced 3MGA can be converted to gallic acid (GA) and 4-carboxy-2-hydroxy-6-methoxy-6-oxohexa-2, 4-dienoate [120, 121], which are further transformed to 4-oxalomesaconate for acetyl-CoA synthesis. The intermediate 3MGA was then converted to 2-pyranone-4, 6-dicarboxylate, which is then converted to acetyl-CoA [122] (Fig. 6). Syringic acid was catalyzed to acetyl-CoA through multiple metabolic steps, which enters TCA cycle for cell growth and product synthesis. In addition to the typical lignin-derived aromatics, some aromatic compounds such as benzene, phenol, benzoate, toluene, and naphthalene can also be catalyzed to catechol [123].

### Degradation of protocatechuic acid and catechol

**Fig. 7:**
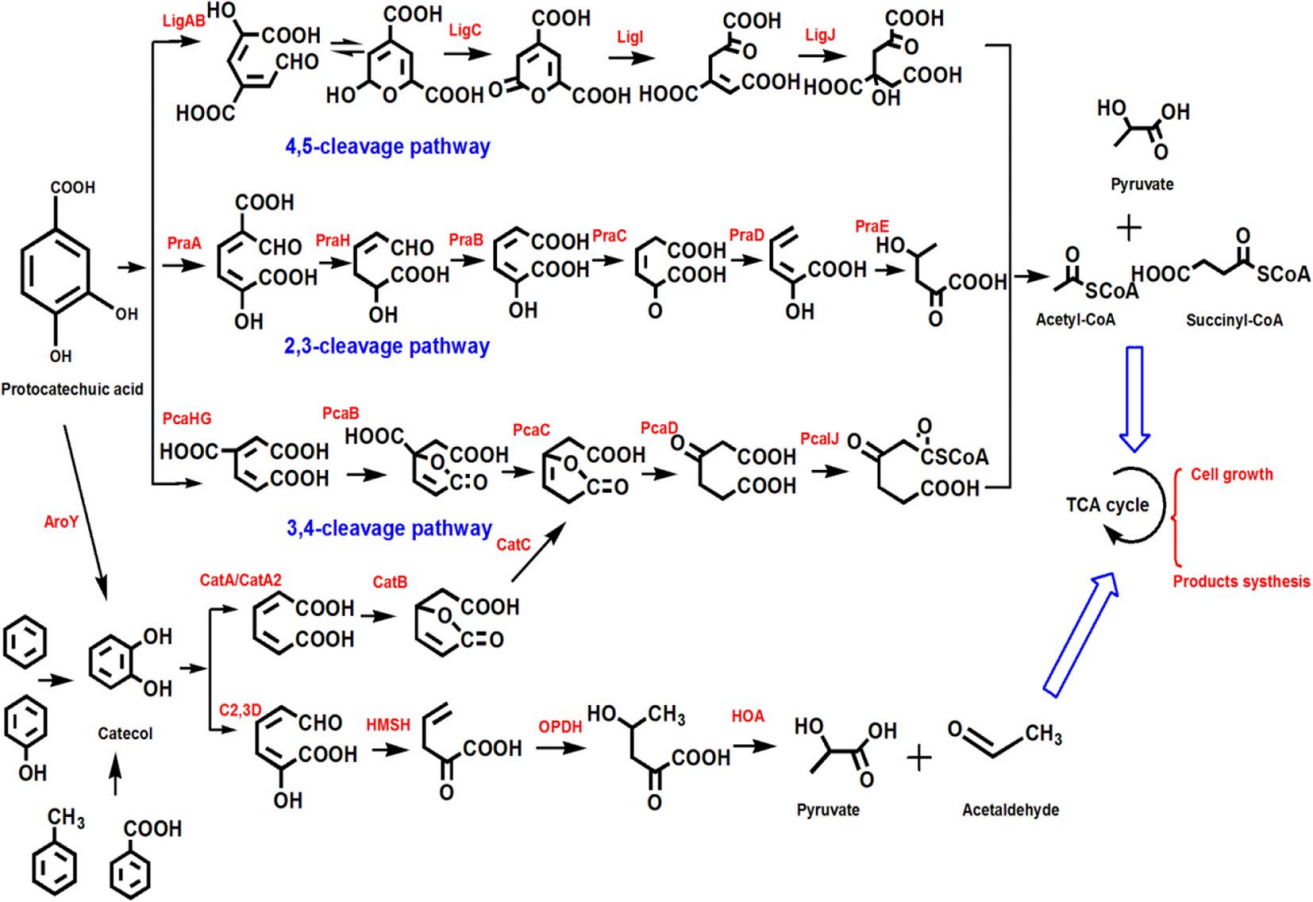
The degradation of protocatechuic acid is categorized as 3, 4-cleavage pathway, 4, 5-cleavage pathway, and 2, 3-cleavage pathway. However, the degradation of catechol is mainly catalyzed by dioxygenases through *ortho*- or *meta*-cleavage pathway [120]. After completing aromatic ring cleavage, the molecules succinate, acetyl-CoA, and pyruvate enter the TCA cycle for cell growth and products’ synthesis

Both protocatechuic acid and catechol are key intermediates in the metabolsim of lignin-based aromatic compounds. The dioxygenase enzymes exhibiting *ortho* (intradiol) or *meta* (extradiol) catalyze the aromatic ring cleavage of protocatechuic acid and catechol [120]. Catechol and protocatechuate are first transformed to *cis, cis*-muconate, and 3-carboxy-*cis, cis*-muconate through ortho-cleavage by O_2_-dependent dioxygenase. Muconates was converted to β-ketoadipate, which reacts with succinyl-CoA and form succinate and β-ketoadipyl-CoA [109]. The final product acetyl-CoA was produced from β-ketoadipyl-CoA and coenzyme A. The meta-cleavage pathways of catechol and protocatechuate are different due to the structural symmetry aspects. The meta-cleavage pathway of protocatechuate was classified into 2, 3 meta-cleavage and 4, 5 meta-cleavage. The 2-hydroxy-5-carboxymuconic semialdehyde was produced in the 2, 3 meta-cleavage pathway of protocatechuate, and finally yield pyruvate and acetyl-CoA. The 2-hydroxy-4-carboxymuconic semialdehyde was produced from 4, 5 meta-cleavage of protocatechuate and eventually generate two pyruvate molecules [124] (Fig. 7). With the cleavage of aromatic ring, the produced intermediates succinate, acetyl-CoA, and pyruvate enter the central metabolism. In short, the metabolism of microorganisms for lignin-based compounds provides an platform for value-added bioproducts’ synthesis.

## Bioconverting lignin to value-added bioproducts by microbial catalysis and metabolic engineering

In the traditional biomass refining process, the complex structure of lignin makes it difficult to be converted into high-value products like carbohydrates. With the elucidation of the structure of lignin and the microbial metabolism of lignin, it is possible to convert lignin into high value-added products through biological methods (Table 4).

**Table 4:** Bioconversion of lignin to value-added products by bacteria

Products	Strains	Carbon source	Yield	Ref
Lipids	*Rhodococcus opacus* DSM 43205	Biomass gasification wastewater	62.8% DCW	[159] (Goswami et al.)
	*R. pyridinivorans* CCZUB16	Alkali lignin	52% DCW	[128] (Chong et al.)
	*R. opacus* DSM 1069	O_2_ pretreated kraft lignin	14.21% DCW	[129] (Wei et al.)
	*R. opacus* PD630	Lignin from combinatorial pretreatment	1.83 g L^−1^	[160] (Liu et al.)
	*R. opacus* Xsp8	Kraft lignin hydrolysate	45.8% DCW	[153] (Kurosawa et al.)
	*R. rhodochrous* ATCC 2198	4-Hydroxybenzoic acid, vanillic acid and glucose	> 40% DCW	[127] (Shields-Menard et al.)
	*R. opacus* DSM 1069 and PD630	4-Hydroxybenzoic acid and vanillic acid	20% DCW	[126] (Kosa, Ragauskas)
	*R. opacus* PD630 *and R. jostii* RHA1 VanA^−^	Alkali-extracted corn stover lignin	39% DCW	[131] (He et al.)
	*Trichosporon cutaneum* ACCC 20271	4-hydroxybenzaldehyde	0.85 g L^−1^	[132] (Hu et al.)
PHAs	*Pseudomonas putida* KT2440	Alkaline-pretreated liquor	34–39% DCW	[136] (Linger et al.)
	*Ralstonia eutropha*	Bagasse hydrolyssate	6.06 g L^−1^	[161] (Yu, Stahl)
	*Azotobacter beijerinicki*	Coir pith	2.4 g L^−1^	[162] (Prabu, Murugesan)
	Engineered *P. putida* A514	Kraft lignin	75 mg L^−1^	[139] (Wang et al.)
	*Oceanimonas doudoroffii*	Lignin and its derivatives	0.2% DCW	[137] (Numata, Morisaki)
	*Cupriavidus basilensis* B-8	Kraft lignin	319.4 mg L^−1^	[138] (Shi et al.)
Vanillin	*R. jostii* RHA045	Wheat straw lignocellulose	96 mg L^−1^	[53] (Sainsbury et al.)
	*Bacillus subtilis*	Ferulic acid	0.89 g L^−1^	[143] (Chen et al.)
	*Streptomyces sannanensis MTCC 6637*	Wheat bran	0.708 g L^−1^	[163] (Chattopadhyay et al.)
	*Shewanella putrefaciens*	Lignin extracted from wheat straw	275 mg L^−1^	[142] (Sharma et al.)
	Engineered *P. putida* KT2440	Ferulic acid	0.86 g g^−1^	[119] (Graf, Altenbuchner)
*Cis,cis*-muconate	*P. putida* KT2440-CJ242	*p*-Coumaric acid	50 g L^−1^	[142] (Sharma et al.)
	Recombinant *C. glutamicum* MA-2	Lignin hydrolysate and Catechol	1.8 g L^−1^ and 85 g L^−1^	[22] (Becker et al.)
	Recombinant *E. coli*	Catechol	59.0 g L^−1^	[149] (Kaneko et al.)
	*P. putida* MA-9	Softwood lignin hydrolysate	13 g L^−1^	[164] (Kohlstedt et al.)
	*Sphingobium* sp. SME257/pTS084	Hardwood lignin hydrolysate	26.8 mg L^−1^	[165] (Sonoki et al.)

### Lipids

**Fig. 8:**
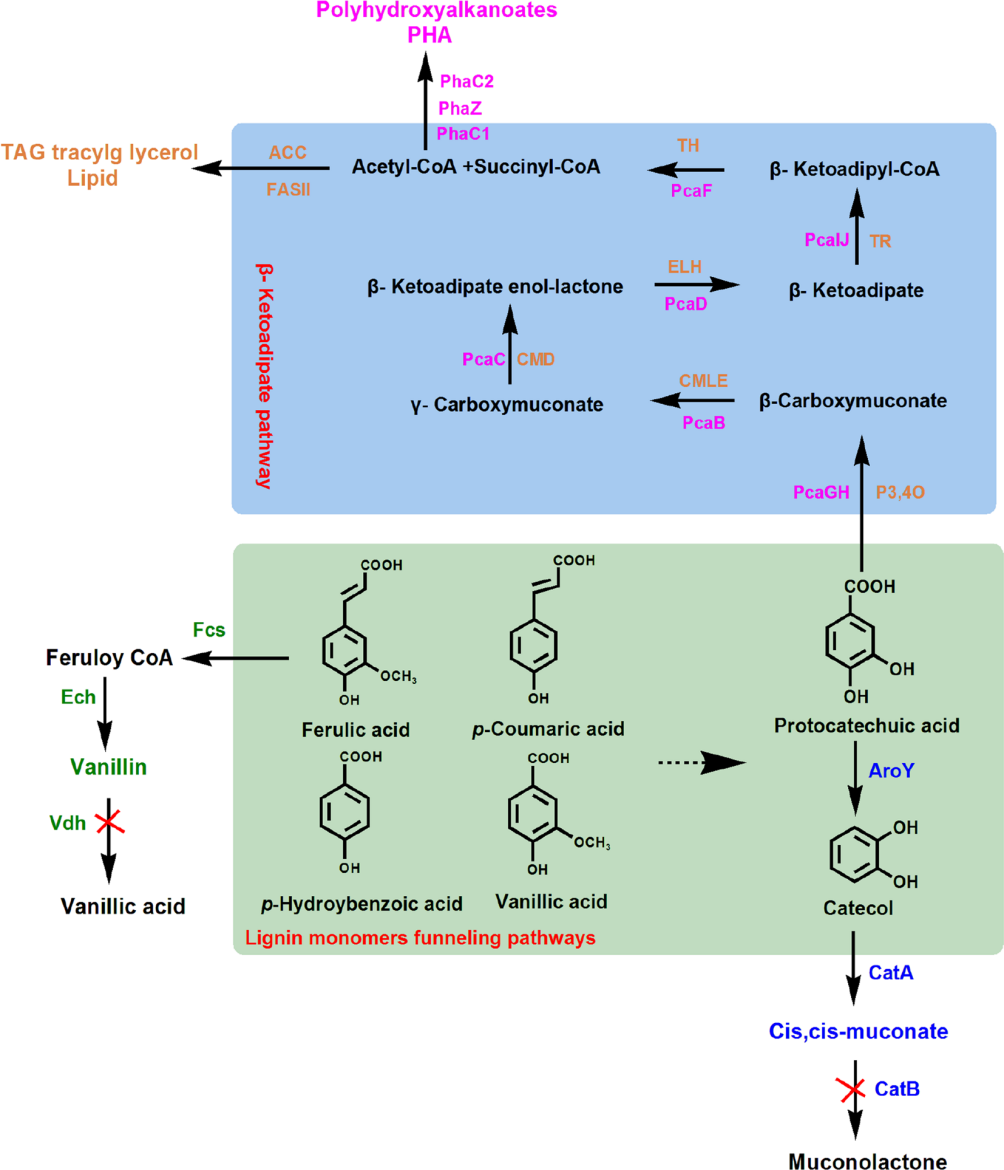
Simple metabolic route for producing valuable compounds from lignin-derived aromatics. Lignin monomers are converted to acetyl-CoA as a final intermediate of protocatechuate metabolism in β-ketoadipate pathway. Lipid and polyhydroxyalkanoates are generated from the significant intermediate of acetyl-CoA via natural catabolic pathways in *Rhodococci opacus* (pink) and *Pseudomonas putida* KT2440 (orange), respectively [58, 126]. *Rhodococcus* sp. RHA1 is engineered to accumulate a significant amount of vanillin from feruilic acid by deleting vanillin dehydrogenase (*Vdh*) gene (green). *Cis, cis*-muconate has been proved to improve its yield in the engineered strain of *P. putida* by deletion of muconate cycloisomerase (*CatB*) gene and insertion of protocatechuate decarboxylase (*AroY*) gene (blue) [59]

The demand for biofuel is expected to grow further due to the increasing global population and depleting fossil resources. Microorganisms can transform lignin to lipids as biofuel. Oleaginous microbes can generate high biomass with more than 20% lipids [125]. The metabolic route of lignin bioconversion to lipid includes four steps (Fig. 8): (1) degradation of low-molecular-weight lignin to its derivatives and other aromatics; (2) catabolism of the aromatic compounds to catechol or protocatechuate; (3) yield acetyl-CoA through aromatic ring cleavage and β-ketoadipate pathway; (4) lipid biosynthesis.

*Rhodococcus* species has been applied for converting lignin and aromatics to lipids; the 4-hydroxybenzoic acid and vanillic acid to triacylglycerols by *R. opacus* DSM 1069 and PD630 strains, which accumulated lipid about 20% of the dry cell weight (DCW) under nitrogen-limiting conditions [126]. *R. rhodochrous* could produce more lipids when cultivated with aromatic compounds and glucose [127]. The alkali, kraft, and ethanol organosolv lignin have also been applied for lipid production with bacteria catalysis. The alkali lignin (4 g L^−1^) could be degraded by *R. pyridinivorans* CCZUB16 with a lipid yield of 52% [128]. The oxygen-pretreated kraft lignin was utilized by *R. opacus* DSM 1069 for lipid production, which was up to 14.21% of CDW and mainly include palmitic (46.9%) and stearic (42.7%) acids [129]. It was found that low-molecular-weight lignin compounds could be assimilated to form lipids more efficiently during the bacterial fermentation. Different strategies have been developed to reduce inhibition and increase lipids yield of microorganism with lignin as substrate. Laccase can synergize with *R. opacus* PD630 for lipid production with insoluble kraft lignin as substrate [130]. The co-fermentation of wild-type *R. opacus* PD630 and engineered *R. jostii* RHA1 VanA^−^ produces higher lipids than single strain fermentation [131]. The yeast *Trichosporon cutaneum* ACCC 20,271 was able to grow with 4-hydroxybenzaldehyde as the sole carbon source, and accumulate 0.85 g L^−1^ of lipid [132]. *Lentinus tigrinus* can accumulate 20% of lipid content in DCW using sunflower seed husks hydrolysates as substrate [133].

### Polyhydroxyalkanoates (PHAs)

Polyhydroxyalkanoates (PHAs) are polyesters synthesized in cells as carbon and energy storage materials in granular forms by various microorganisms under nutrient imbalance conditions. With excellent biocompatibility and biodegradability, PHAs have been widely used in biomedicine, bioplastics, and nanotechnology [134, 135]. In nature, many bacteria have developed metabolic pathways for converting lignin to PHAs with short-, medium-, or long-chain length (scl, mcl, and lcl). The lignin derivatives can be metabolized to acetyl-CoA for PHA synthesis (Fig. 8).

Currently, the PHA productions from lignin or lignin-related aromatic compounds have been achieved in various bacteria. The aramatics of *p*-coumarate and ferulate can be converted to mcl-PHA by *P. putida* KT2440, and comparable mcl-PHA was also accumulated with APL as substrate [136]. The marine bacterium *Oceanimonas doudoroffii* can synthesize PHA from lignin and its derivatives such as sinapinic acid and syringic acid [137]. The untreated kraft lignin (5 g L^−1^) was converted to PHA (128 mg L^−1^) by *Cupriavidus basilensis* B-8, and PHA concentration was up to 319.4 mg L^−1^ through fed batch fermentation [138]. System biology approach was developed to enhance PHA production from kraft lignin with *P. putida* A514, and the PHA content reached 73.5% (DCW), in which the dye peroxidase-based enzymatic system was optimized, and enzymes were overexpressed to promote central metabolism, and the β-oxidation of fatty acids were up-regulated to maximize carbon flux into PHA synthesis [139]. Besides improving the lignin utilization capability of related bacteria, lignin pretreatments were also applied to improve PHA production. *P. putida* KT2440 accumulate higher production of PHA from lignin pretreated with H_2_SO_4_ and NaOH [140]. The generated PHA can be converted to diverse chemicals precursors like alkenoic acids and hydrocarbons, which indicated that the lignin can be converted to biomaterials, chemical precursors, and fuel-range hydrocarbons.

### Vanillin

As lignin has the unique aromatic structure, some value-added intermediates can be accumulated in the process of lignin degradation and metabolism. Vanillin (4-hydroxy-3-methoxybenzaldehyde) is one of the most important aromatic compounds and has been widely applied to food, cosmetics, pharmaceutical, and other industries [141]. Vanillin is usually extracted from natural plant or synthesis through chemical synthesis, and biosynthesis of vanillin with lignin as a feedstock is a clean and promising method. In the metabolic process of lignin, vanillin can be released from lignin through depolymerization or produced from ferulic acid through microbial catalysis (Fig. 8). A microbial fuel cell system was designed for depolymerizing lignin and produces vanillin through H_2_O_2_-mediated oxidative reaction [142]. Vanillin can be produced from ferulic acid with *Bacillus subtilis*, *Streptomyces* sp., and *Amycolatopsis* sp. [143].

Metabolic engineering has been developed to improve the yield of vanillin in microorganisms. It has been reported that vanillin accumulation in *Amycolatopsis* sp. ACTT 39,116 was achieved by the deletion of *vdh* gene, which encodes NAD-dependent vanillin dehydrogenase for converting vanillin to vanillic acid, and the mutant strain produced 6.5 mM vanillin with 2 mM ferulic acid as substrate [144]. *P. putida* KT2440 was optimized to convert 86% of ferulic acid to vanillin with low by-product, in which strong tac promoter was applied to enhance the expression of *fcs* and *ech* [117]. With excellent antimicrobial, antioxidant properties, and low toxicity, *p*-hydroxybenzoic acid and pyrogallol are produced from lignin and its derivative [145, 146].

### *Cis, cis*-muconate (*cis, cis*-MA)

*Cis, cis*-muconate (*cis, cis*-MA) is a six-carbon di-unsaturated dicarboxylic acid and a direct precursor for adipic acid and terephthalic acid, which are mainly used to produce polymers including nylon, polyurethane, and polyethylene terephthalate (PET) [147]. *Cis, cis*-MA has been conventionally produced through chemical synthesis using petroleum-based feedstocks and generating toxic intermediates. Therefore, production of *cis, cis*-MA from lignocellulosic biomass provides a feasible alternative strategy to alleviate the environmental issues in chemical synthesis. Lignin-based aromatics was converted to *cis, cis*-MA by microbial catalysis, which is a crucial intermediate of aromatics metabolism. The *cis, cis*-MA was accumulated and secreted into the culture broth when its degradation route was disrupted (Fig. 8). The engineered strains such as *P. putida*, *Amycolatopsis* sp, *E. coli*, and *Corynebacterium glutamicum* have been reported to produce high MA yields from lignin-based aromatics. The production of *cis, cis*-MA from *p*-coumaric acid with the engineered *P. putida* KT2440 reached 50 g L^−1^, in which two associated proteins (EcdBD) were co-expressed and a global regulator of carbon catabolite repression was eliminated [148]. Becker et al. [22] engineered the *C. glutamicum* MA-2 strain with the elimination of muconate cycloisomerase (*catB*) and overexpression of catechol-1 and 2-dioxygenase (*catA*), which produces respective 85 g L^−1^ and 1.8 g L^−1^ *cis, cis*-MA from catechol and hydrothermal pretreated softwood lignin. Similar *E. coli* was constructed by expressing the catA gene from *P. putida* mt-2 and produced 59 g L^−1^ *cis, cis*-MA from catechol with a molar yield of 100% in a fed-batch fermentation [149].

The dicarboxylic acids like pyridine-2, 4-dicarboxylic acid (2, 4-PDCA) and pyridine-2, 5-dicarboxylic acid (2, 5-PDCA) can also be produced from lignin and serve as building blocks for polyamides and polyesters [150]. The bacterium *R. jostii* RHA1 metabolize lignin through the β-ketoadipate pathway. The metabolic pathways of *R. jostii* RHA1 were engineered by insertion of genes ligAB-encoding protocatechuate 4,5-dioxygenase and protocatechuate 2,3-dioxygenase, and 80 mg L^−1^ 2,4-PDCA and 125 mg L^−1^ 2,5-PDCA were produced when cultured on minimal media containing 1% wheat straw lignocellulose [54].

## Conclusions

Lignin is the most abundant aromatic biopolymer in nature and an excellent substrate for value-added bioproducts synthesis. Bioprocessing with microorganisms and enzymes is a clean and efficient method for lignin utilization, while the low efficiency of lignin valorization is a challenge in the process. The pretreatment can break lignin to small fragments, which can improve the bioavailability of which to microorganisms [151]. The oxidase secreted by microorganims are crucial for lignin degradation, which has been applied to improve lignin the bioavailability and depolymerization in vitro [152]. The cell growth inhibition by lignin-derived aromatics is another issue in lignin bioprocessing and utilization. The strategies including microorganisms acclimation and fed-batch operation have been applied to mitigate the inhibitory effects of aromatic compounds [153]. The elucidation of the pathway for lignin degradation and metabolism and its aromatic compounds provide an platform for lignin depolymerization and biotransformation into value-added products through metabolic engineering. Further research on microbial metabolic engineering and industrial process scale-up are still required to realize the efficient lignin depolymerization and value-added products’ biosynthesis.

## Data Availability

Not applicable.

## Abbreviations

MnP: Manganese peroxidase
LiP: Lignin peroxidase
VP: Versatile peroxidase
DyP: Dye-decolorizing peroxidase
TCA: Tricarboxylic acid cycle
PPO: Polyphenol oxidase
H_2_O_2_: Hydrogen peroxide
APL: Alkaline pretreated liquor
NAD(P)H: Nicotinamide adenine dinucleotide phosphate
ATP: Adenosine triphosphate
ABTS: 2,2′-Azinobis-(3-ethylbenzothiazoline-6-sulfonate)
HAA: 3-Hydroxyanthranilic acid
HBT: 1-Hydroxybenzotriazole
VA: Veratryl alcohol
GS-HPV: α-Glutathionyl-β-hydroxypropiovanillone
PCB: Polychlorinated biphenyls
DDVA: 5, 5′-Dehydrodivanillate
5CVA: 5-Carboxyvanillic acid
pHCS: *p*-Hydroxycinnamoyl CoA synthetase
pHCHL: *p*-Hydroxycinnamoyl CoA hydratase/lyase
BADH: Benzaldehyde dehydrogenase
*fcs*: Feruloyl-CoA synthetase
*ech*: Enoyl-CoA hydratase/aldolase
*vdh*: Vanillin dehydrogenase
*vanAB*: Vanillate-*O*-demethylase
3MGA: 3-*O*-Methylgallate
DesA: Tetrahydrofolate-dependent *O*-demethylase
GA: Gallic acid
DCW: Dry cell weight
PHAs: Polyhydroxyalkanoates
*cis, cis*-MA: *cis, cis*-Muconate
PET: Polyethylene terephthalate
*catB*: Muconate cycloisomerase
*catA*: Catechol-1,2-dioxygenase
2,4-PDCA: Pyridine-2,4-dicarboxylic acid
2,5-PDCA: Pyridine-2,5-dicarboxylic acid
